# WTAP Accelerates Exhaustion of CD8^+^ T Cells and Progression of Hepatocellular Carcinoma by Promoting m6A Modification and Translation of PD1 mRNA

**DOI:** 10.1155/mi/6217272

**Published:** 2025-06-18

**Authors:** Rong Li, Shunle Li, Hua Li, Bingli Liu, Zimu Wang, Huanqin Lei, Yuting Li, Lijuan Jia, Junhui Li, Hongwei Lu, Meng Xu

**Affiliations:** ^1^Department of Anesthesiology, The Second Affiliated Hospital of Xi'an Jiaotong University, Xi'an Jiaotong University, Xi'an, Shaanxi, China; ^2^Department of General Surgery, The Second Affiliated Hospital of Xi'an Jiaotong University, Xi'an Jiaotong University, Xi'an, Shaanxi, China; ^3^Xi'an Jiaotong University Health Science Center, Xi'an Jiaotong University, Xi'an, Shaanxi, China

**Keywords:** liver cancer, N6-methyladenosine, PD1, T cell exhaustion, WTAP

## Abstract

The N6-methyladenosine (m6A) methylase WTAP has been identified as a proto-oncogene in multiple cancers, including hepatocellular carcinoma (HCC). Interestingly, although WTAP expression does not differ between normal liver and HCC tissues or across different stages of HCC, patients with higher WTAP expression exhibit significantly shorter median survival times (MSTs). Here, we found that WTAP was upregulated in tumor-infiltrating CD8^+^ T cells, which were more enriched in HCC patients compared to the controls. HCC patients also displayed higher PD1 levels and a greater proportion of exhausted CD8^+^ T cells (TCF^+^ PD1^+^). Moreover, WTAP promoted PD1 expression and suppressed the proliferation and immune activity of CD8^+^ T cells. In the co-culture system, WTAP-overexpressing CD8^+^ T cells enhanced the malignancy of HCC cells. Notably, WTAP silencing further augmented the boosting effect of PD1 silencing on CD8^+^ T cell immune activity and strengthened its inhibitory effect on HCC cell growth. As an m6A “writer”, WTAP increased the m6A level of PD1 mRNA, thereby promoting YTHDF1-mediated translation of PD1. Finally, in the HuNSG xenograft tumor model, WTAP knockdown not only alleviated CD8^+^ T cell exhaustion and inhibited tumor progression but also synergistically enhanced the antitumor efficacy of anti-PD1 therapy. In conclusion, WTAP promoted CD8^+^ T cell exhaustion and HCC progression by facilitating the m6A modification and translation of PD1 mRNA.

## 1. Introduction

Hepatocellular carcinoma (HCC) is a leading cause of cancer-related death worldwide [1]. Most HCC cases are diagnosed at advanced stages, often due to delayed diagnosis and rapid disease progression, resulting in a 5-year survival rate of less than 20% [2]. Despite notable progress in the diagnosis and treatment of HCC over the past few years, the incidence and mortality of HCC still accelerate rapidly [3, 4]. Therefore, it is urgently needed to develop new therapeutic approaches for advanced HCC.

A complex biological regulatory network exists between the immune system and malignancies [5, 6]. Tumor-specific cytotoxic CD8^+^ T lymphocytes are known to recognize tumor antigens and inhibit tumor growth [7]. However, sustained stimulation of tumor antigens eventuates in dysfunction of immunocompetent CD8^+^ T cells, manifested by a cessation of proliferation, cytokine production, and immune activity [8, 9]. Programed cell death protein 1 (PD-1) is an immune checkpoint molecule expressed by chronically activated CD8^+^ and CD4^+^ T cells [10, 11]. PD-1 is copious in circulating CD8^+^ T and tumor-infiltrating lymphocytes in cancer patients [12, 13], which predicts poor prognosis in many cancers, including HCC [14].

N6-methyladenosine (m6A) modification is the preponderant post-transcriptional modification in mammals [15]. It is catalyzed by m6A methyltransferases (mainly METTL3/14 and WTAP), removed by m6A demethylases (mainly ALKBH5 and FTO), and recognized by m6A readers (mainly YTHDF family, YTHDC family, IGFBP family, and HNRNP family), playing an important role in various biological processes [16]. M6A methylation regulates mRNA transport, splicing, stability/decay, and translation, and is involved in many diseases by affecting cell cycle, aging, apoptosis, and fate [17, 18]. WTAP is a nuclear methyltransferase, famous as the chaperone of Wilms tumor 1 (WT1) [19]. It plays a crucial role in regulating X-chromosome inactivation, alternative splicing, and the cell cycle [19, 20]. Growing evidence points to a strong link between WTAP and cancer progression. WTAP acts as an oncogene in osteosarcoma, B-cell lymphoma, and ovarian cancer [21–23]. WTAP has also been reported to be overexpressed in HCC, promoting tumor growth by facilitating m6A of ETS1 mRNA [24]. However, the role of WTAP in regulating HCC progression through modulation of CD8^+^ T cell immune activity remains unclear.

In this study, we demonstrated that WTAP was upregulated in tumor-infiltrating CD8^+^ T cells and was significantly positively correlated with PD1 expression. We then investigated whether WTAP affected the expansion and immune function of CD8^+^ T cells by regulating PD1 expression and its influence on HCC cell malignancy.

## 2. Materials and Methods

### 2.1. Patient Selection and Tumor Specimens

Paired normal and HCC tissues were obtained from 124 HCC patients who underwent tumor resection surgery at the Second Affiliated Hospital of Xi'an Jiaotong University. All patients had not received systemic anticancer or immunosuppressive therapy for at least 3 months before surgery. This study was approved by the Ethics Committee of Xi'an Jiaotong University (XJTU-2021045) and adhered to the standards set in the Declaration of Helsinki.

### 2.2. CD8^+^ T Cells Isolation

The EasySep Direct Human CD8^+^ T-Cell Isolation Kit (StemCell Technologies, Canada) was used for CD8^+^ T cell isolation from peripheral blood mononuclear cells (PBMCs) of HCC patients. CD8^+^ T cells were incubated with anti-CD3/CD28 antibodies for 2 days to activate T cells.

### 2.3. Cell Culture and Transfection

HCC cell lines (HCCLM3 and MHCC97H) were purchased from SUNNCELL (Wuhan, Hubei, China). DMEM (Gibco, Rockville, MD, USA) medium supplemented with FBS (10%, Gibco) and P/S (1%, 100 μL/mL) was used for cell culture at 37°C in a 5% CO_2_ atmosphere.

WTAP overexpression vector (pcDNA3.1-WTAP) was obtained from Sangon Biotech (Shanghai, China). GenePharma (Shanghai, China) synthesized WTAP siRNA, YTHDF1 siRNA and NC siRNA. Lentivirus-mediated WTAP short hairpin RNA (sh-WTAP) vectors were constructed by GeneChem (Shanghai, China). Anti-PD-1 (#BE0188) and control IgG (#BE0297) were purchased from BioXcell (NH, USA). Lipofectamine 3000 (Invitrogen Corporation, CA, USA) was used for cell transfection.

### 2.4. Flow Cytometry Analysis

Normal liver tissue and HCC tumor-infiltrating T cells were stained with anti-TCF1, anti-CD4, anti-CD8a, and anti-PD1 antibodies. Flow cytometry data were acquired with an LSR-II instrument, and cells were sorted on an Aria II sorter. FlowJo software (TreeStar) was used for data analysis.

### 2.5. Western Blot Analysis

Total protein extraction was conducted using RIPA (Beyotime, Shanghai, China), and protein concentration was determined using a Pierce BCA Protein Assay Kit (Thermo). Approximately 50 μg of protein samples were separated by SDS-PAGE and was transferred onto PVDF membranes. Membranes were then incubated with primary antibodies against WTAP (#56501, Cell Signaling Technology [CST], Boston, MA, USA); PD1 (ab52587, Abcam, Cambridge, UK); YTHDF1 (ab290749, ab52587); and YTHDF2 (ab220163, ab52587) after blocking with nonfat milk (5%). Secondary antibodies were applied for membrane incubation, and band visualization was performed using an enhanced chemiluminescence kit (Beyotime). Band intensities were analyzed using ImageJ software.

### 2.6. RT-qPCR

Total RNA extraction was performed using TRIzol reagent (Takara, Dalian, China). cDNA was obtained using a PrimeScript RT reagent Kit (Takara). RT-qPCR was carried out using a TB Green Premix Ex Taq kit (TaKaRa). The primers used were as follows: WTAP-F-5′-TTC CCA AGA AGG TTC GAT TG-3′, WTAP-R-5′-TGC AGA CTC CTG CTG TTG TT-3′; PD1-F-5′-ACC TGG GTG TTG GGA GGG CA-3′, PD1-R-5′-GGA GTG GAT AGG CCA CGG CG-3′; GAPDH-F-5′-GGA GCG AGA TCC CTC CAA AAT-3′, GAPDH-R-5′- GGC TGT TGT CAT ACT TCT CAT GG-3′. GAPDH served as the internal control. Gene expressions were calculated using the 2^−ΔΔct^ method.

### 2.7. ELISA

The levels of perforin, IFN-γ, granzyme-B, and TNF-α produced by CD8^+^ T cells were determined using corresponding commercial ELISA kits (eBioscience, CA, USA) following the manufacturer's guidelines.

### 2.8. Methylated RNA Immune-Precipitation (MeRIP)-qPCR Assay

Fragmented RNA samples from CD8^+^ T cells were immunoprecipitated using the Magna MeRIP m6A Kit (Millipore, Boston, MA, USA) and anti-m6A antibody (ab286164, Abcam) following the instructions of the manufacturer. The level of m6A-modified PD1 was detected by RT-qPCR.

### 2.9. Cell Apoptosis

An Annexin V-FITC/PI apoptosis detection kit (Beyotime) was used for cell apoptosis detection. HCCLM3 and MHCC97H cells were collected, washed with PBS, and sequentially incubated with Annexin V (5 μL) and PI (1 μL) for 10 min. Apoptotic cells were measured using a flow cytometer (Becton Dickinson, San Jose, CA, USA).

### 2.10. Transwell Assay

Cell invasion of HCCLM3 and MHCC97H cells was assessed using 8 μm microporous filters coated with Matrigel (Becton, Bedford, MA, USA). 1 × 10^4^ cells were added to the upper chamber with 200 μL of serum-free DMEM medium, and the bottom chamber was supplemented with DMEM medium containing 10% FBS. After 24 h, cells on the lower surface of the filter were fixed with formaldehyde and stained with hematoxylin for 20 min. Finally, cells in five randomized regions were photographed and counted.

### 2.11. Colony Formation Assay

HCCLM3 or MHCC97H cells (5 × 10^4^) were seeded in 6-well plates. After treatment, cells were fixed in paraformaldehyde (4%) for 20 min at 4°C, followed by staining with crystal violet (0.25%) for 20 min at room temperature. Cells were then washed with ddH_2_O to remove excess staining solution. Colony formation was observed using a light microscope, and images were captured. A colony was defined as consisting of at least 50 cells.

### 2.12. RNA Immunoprecipitation (RIP)

RIP assay was performed using the Magna RIP Kit (17–700, Millipore, MA) following the manufacturer's instructions. Briefly, anti-WTAP, anti-YTHDF1, anti-YTHDF2, and IgG antibodies were incubated with protein A/G magnetic beads for 30 min at room temperature. Cell lysates (more than 2 × 10^7^ cells per sample) were collected and incubated with the bead-antibody conjugates at 4°C overnight. The beads were then treated with proteinase K for 30 min to remove proteins. The RNA in the immune complexes was detected using RT-qPCR.

### 2.13. EdU Assay

HCCLM3 or MHCC97H cells (6 × 10^3^/well) were seeded in a 96-well plate for 24 h, and EdU reagent (50 μM, 100 μL) was added. Two hours later, cells were sequentially treated with paraformaldehyde (4%, 30 min), Triton X-100 (0.5%, 10 min), and 1x Apollo Stain Buffer (0.5 h). After staining with DAPI, cells were photographed under a fluorescence microscope (Nikon, Japan).

### 2.14. Humanized Mouse Generation

NSG mice were purchased from VITALSTAR (Beijing, China) and raised under SPF conditions. CD34^+^ hematopoietic stem cells were obtained from the human fetal liver tissues as previously described [25]. NSG mice (1–3 day old) were irradiated (1 Gy) and injected intravenously with CD34^+^ human hematopoietic stem cells (2 × 10^5^ cells/mouse). The reconstitution of the human immune system was analyzed using peripheral blood from humanized NSG (HuNSG) mice 3 months after injection and before the start of the experiment.

### 2.15. In Vivo Tumor Growth

This study was approved by the Animal Care and Use Committee of the Xi'an Jiaotong University. HuNSG mice were randomly divided into 5 groups (*n* = 8): (1) control group: 100 μL of PBS containing 2 × 10^5^ HCCLM3 cells were injected into the right flank of the mice; (2) NC shRNA group: 100 μL of PBS containing 2 × 10^5^ HCCLM3 cells infected with Lv-sh-NC were injected into the right flank of the mice; (3) sh-WTAP group: 100 μL of PBS containing 2 × 10^5^ HCCLM3 cells infected with Lv-sh-WTAP were injected into the right flank of the mice; (4) anti-PD1 group: 100 μL of PBS containing 2 × 10^5^ HCCLM3 cells were injected into the right flank of the mice, followed by Opdivo (100 μg) injection through the tail vein for 2 weeks (three times a week); and (5) combination group: 100 μL of PBS containing 2 × 10^5^ HCCLM3 cells infected with Lv-sh-WTAP were injected into the right flank of the mice, followed by Opdivo (100 μg) injection through the tail vein for 2 weeks (three times a week). Twenty-eight days after injection, tumor tissues and spleen tissues were isolated for subsequent studies.

### 2.16. scRNA-Seq Data Processing and Analysis

The single-cell RNA sequencing data comprising CD45^+^ immune cells isolated from five distinct immune-related anatomical sites in HCC patients were obtained from the GEO dataset GSE140228. Single-cell RNA sequencing data were processed using the Seurat package. Initial quality control removed cells with fewer than 3 detected genes (minimum cells = 3), UMI counts below 500, gene counts under 250 (n gene > 250), or mitochondrial content exceeding 10% (mitoratio < 0.1). The SCTransform normalization and integration workflow was applied, followed by UMAP dimensionality reduction. Cell clusters were annotated via singleR to characterize CD8^+^ T cell heterogeneity.

### 2.17. Statistical Analysis

SPSS 22.0 was used for statistical analyses, and data were presented as mean ± SD. Each experiment was independently repeated at least three times. Statistical significance was assessed using Student's *t*-test and one-way ANOVA, followed by Tukey's post hoc test. *p* < 0.05 indicated statistically significant.

## 3. Results

### 3.1. High Levels of WTAP Were Associated With Shorter Survival in HCC Patients

**Figure 1:**
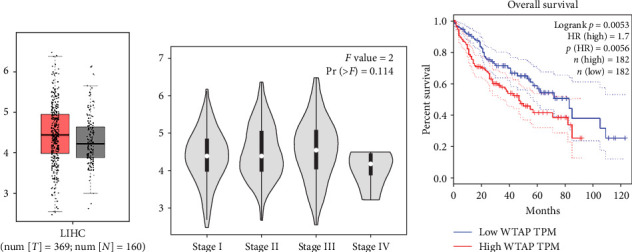
High expression of WTAP was associated with shorter survival in HCC patients. (A) The GEPIA online database was used to analyze the expression level of WTAP in HCC tissues and adjacent normal tissues. (B, C) The GEPIA online database was used to assess the relationship between the level of WTAP and the clinical stage and overall survival rate of HCC patients.

The GEPIA online database was used to analyze the expression pattern of WTAP in liver cancer and normal tissues, as well as its correlation with the overall survival rate and clinical stage of liver cancer patients. The data showed that WTAP levels did not differ significantly between liver cancer and para-carcinoma tissues (Figure 1A). Moreover, no prominent difference was observed in WTAP expression among liver cancer specimens of different stages (Figure 1B). However, higher levels of WTAP predicted a shorter median survival time (MST) in liver cancer patients (<50 vs. >80 months, *p*=0.0053) (Figure 1C).

### 3.2. WTAP Was Upregulated in Tumor-Infiltrating Exhausted CD8^+^ T Cells

**Figure 2:**
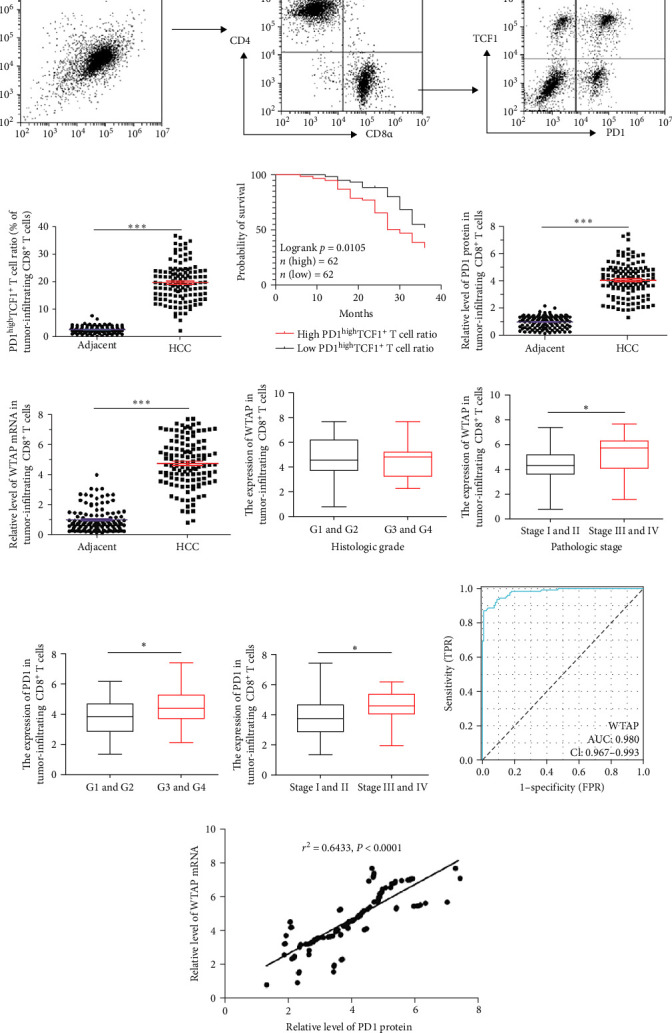
WTAP was highly expressed in tumor-infiltrating exhausted CD8^+^ T cells. (A) Representative flow cytometric plots showing the sorting process of CD8^+^ T cells and PD1^high^TCF1^+^ CD8^+^ T cells. (B) Comparison of the frequencies of PD1^high^TCF1^+^ T cells among CD8^+^ T cells across paracancerous and tumor tissues of HCC patients. (C) The Kaplan–Meier survival analysis. (D) The protein expression of PD1 in tissue-infiltrating CD8^+^ T cells was detected using western blot analysis. (E) RT-qPCR was performed to determine the mRNA level of WTAP in tissue-infiltrating CD8^+^ T cells. (F–I) Expression of WTAP and PD-1 in different clinical subtypes of HCC samples. (J) ROC analysis for WTAP. (K) Correlation between WTAP and PD-1 levels was analyzed by using Pearson's correlation test. *N* = 124. *⁣*^*∗*^*p* < 0.05 and *⁣*^*∗∗∗*^*p* < 0.001.

It has been reported that co-expression of PD1 and TCF1 by CD8^+^ T cells is indicative of T cell exhaustion. We first screened for tissue-infiltrating T cells from adjacent and tumor tissues, and further isolated CD8^+^ T cells (Figure 2A). Subsequently, the proportion of PD1^high^TCF1^+^ T cells in the para-cancer and tumor groups was analyzed. The proportion of PD1^high^TCF1^+^ CD8^+^ T cells was significantly higher in tumor tissues compared to the para-cancer group (Figure 2B). Based on the median proportion of tumor-infiltrating PD1^high^TCF1^+^ CD8^+^ T cells, 124 HCC patients were divided into low-ratio and high-ratio groups. The Kaplan–Meier curve showed a significantly higher 3-year survival rate among patients in the low-ratio group compared to the high-ratio group (Figure 2C). Moreover, PD1 protein levels and WTAP mRNA levels in tissue-infiltrating CD8^+^ T cells were increased in the HCC group compared to the para-cancer group (Figure 2D,E).  Table 1 lists the relationships between the expression levels of WTAP in tissue-infiltrating CD8^+^ T cells and the clinicopathologic characteristics of 124 HCC patients. High expression of WTAP and PD1 was associated with advanced pathological stage in HCC, while elevated PD1 expression additionally correlated with higher histological grade (Figure 2F–I). However, neither WTAP nor PD1 expression levels showed significant associations with patient age or gender (Figure S1A–D). ROC curve analysis was used to evaluate the predictive value of WTAP in the diagnosis of liver cancer. The area under the curve (AUC) of the WTAP was 0.98 (Figure 2J). Pearson's correlation analysis indicated a strong positive correlation between PD1 and WTAP levels (Figure 2K). Furthermore, analysis of the TISIDB database revealed that the expression levels of both WTAP and PD-1 showed significant positive correlations with the abundance of activated CD8^+^ T cells (Figure S1E,F). Additionally, scRNA-seq data comprising CD45^+^ immune cells isolated from five distinct immune-related anatomical sites in HCC patients were downloaded from the GSE140228 dataset. Figure S1G,H illustrates the differential distribution patterns of TCF1 and WTAP among distinct CD8^+^ T cell subsets within the tumor immune microenvironment of HCC. Quantitative analysis revealed that TCF1^+^ and WTAP^+^ cells accounted for 11.8% and 31.6% of PD-1^+^ CD8^+^ T cell subpopulations, respectively (Figure S1I,J).

### 3.3. WTAP Overexpression Suppressed CD8^+^ T Cell Function and Upregulated PD1 Expression

**Figure 3:**
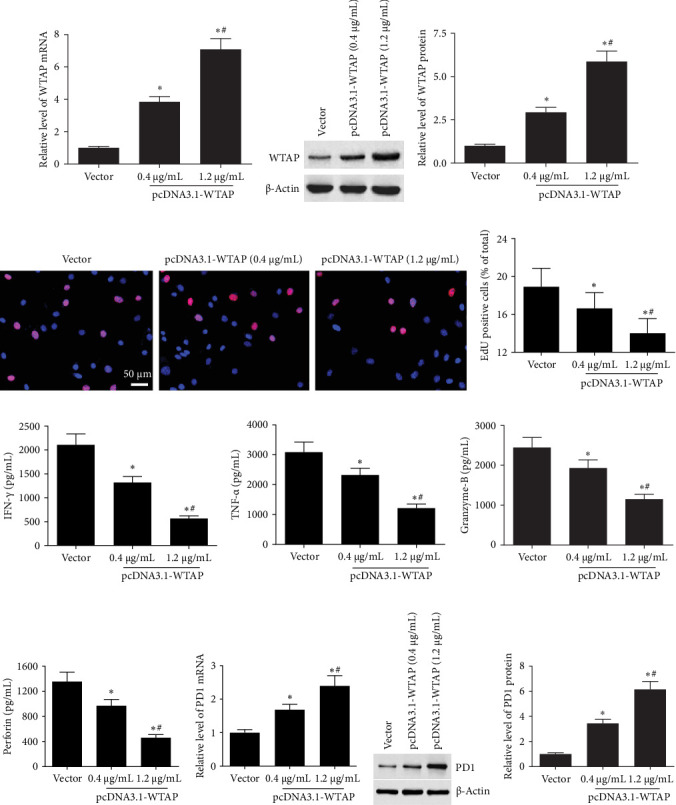
Overexpression of WTAP suppressed cell function and increased PD1 expression in CD8^+^ T cells. 0.4 and 1.2 μg/mL pcDNA-WTAP were transfected into CD8^+^ T cells, and then CD8^+^ T cells were activated by incubating with anti-CD3/anti-CD28 antibodies for 48 h. (A) The mRNA level of WTAP was measured with RT-qPCR. (B, C) Western blotting was conducted to detect the protein expression of WTAP. (D, E) EdU assay was used to assess cell proliferation of CD8^+^ T cells. (F–I) ELISA assay was performed to analyze the secretion levels of TNF-α, IFN-γ, perforin, and granzyme-B. (J–L) The mRNA and protein levels of PD1 in CD8^+^ T cells were detected. *⁣*^*∗*^*p* < 0.05 compared with the vector group. ^#^ *p* < 0.05 compared with the 0.4 μg/mL group.

To explore the role of WTAP in CD8^+^ T cell function, different concentrations of WTAP overexpression vectors were transfected into CD8^+^ T cells, which were then activated by anti-CD3/anti-CD28 stimulation. After transfection with pcDNA-WTAP, WTAP expression was notably elevated in a concentration-dependent manner (Figure 3A–C). Some cytokines, including TNF-α, IFN-γ, perforin, and granzyme-B, play key roles in antitumor response. Overexpression of WTAP significantly suppressed CD8^+^ T cell proliferation and cytokine secretion (including perforin, IFN-γ, granzyme-B, and TNF-α) (Figure 3D–I). Additionally, ectopic expression of WTAP notably increased PD1 expression at both the transcription and translation levels (Figure 3J–L).

### 3.4. Silencing WTAP Promoted CD8^+^ T Cell Function and Suppressed PD1 Expression

**Figure 4:**
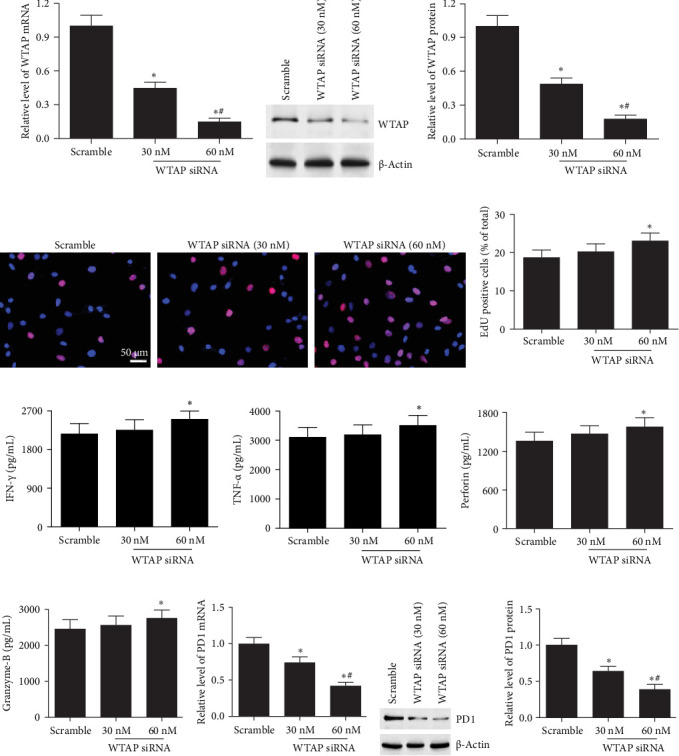
Silencing WTAP promoted cell function and suppressed PD1 expression in CD8^+^ T cells. 30 and 60 nM WTAP siRNA were transfected into CD8^+^ T cells, and then CD8^+^ T cells were activated by incubating with anti-CD3/anti-CD28 antibodies for 48 h. (A–C) The mRNA and protein expression of WTAP was detected using RT-qPCR and western blotting. (D, E) EdU assay was conducted to evaluate cell proliferation of CD8^+^ T cells. (F–I) ELISA assay was used to analyze the secretion levels of TNF-α, IFN-γ, perforin, and granzyme-B. (J–L) The mRNA and protein levels of PD1 in CD8^+^ T cells were measured. *⁣*^*∗*^*p* < 0.05 compared with the scramble group. ^#^ *p* < 0.05 compared with the 30 nM group.

Next, 30 nM or 60 nM WTAP siRNA was used to inhibit WTAP expression in CD8^+^ T cells. Treatment with WTAP siRNA significantly reduced WTAP expression in a dose-dependent manner (Figure 4A–C). Moreover, CD8^+^ T cells treated with 60 nM WTAP siRNA, but not 30 nM, showed significantly increased cell proliferation and cytokine secretion levels (Figure 4D–I). Furthermore, silencing WTAP markedly decreased PD1 expression in CD8^+^ T cells (Figure 4J–L).

### 3.5. WTAP Overexpression in CD8^+^ T Cells Promoted Malignant Progression of HCC Cells

**Figure 5:**
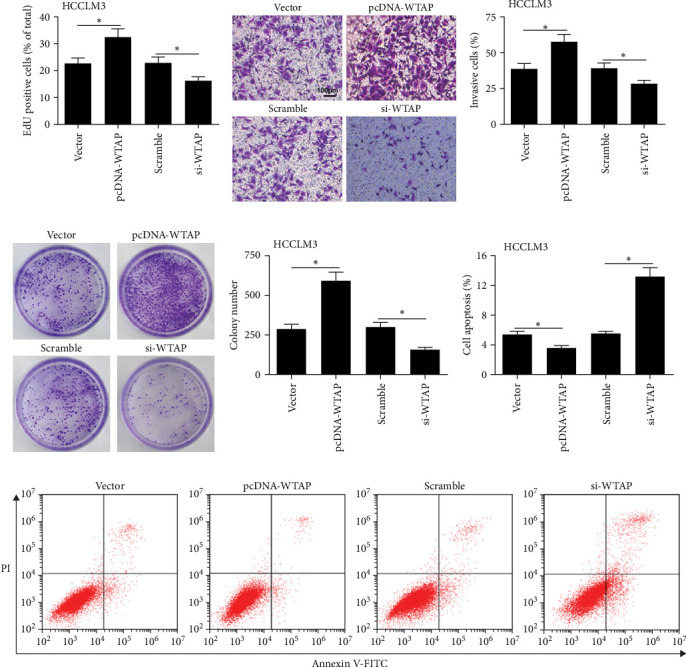
Overexpression of WTAP in CD8^+^ T cells promoted malignant progression of HCC cells. pcDNA-WTAP or WTAP siRNA were transfected into CD8^+^ T cells for 24 h, which were then co-cultured with HCCLM3 cells for 24 h. (A) EdU assay was conducted to evaluate cell proliferation of HCCLM3 cells. (B, C) Cell invasion of HCCLM3 cells was analyzed using the Transwell assay. (D, E) Colony formation assay was used to determine colony formation in HCCLM3 cells. (F, G) Flow cytometry was performed to detect HCCLM3 cell apoptosis. *⁣*^*∗*^*p* < 0.05.

To explore the effect of CD8^+^ T cells overexpressing or silencing WTAP on the behavior of HCC cells, pcDNA-WTAP, or WTAP siRNA was transfected into CD8^+^ T cells, which were then co-cultured with HCCLM3 or MHCC97H cells. Compared to the vector group, overexpressing WTAP in CD8^+^ T cells significantly facilitated cell proliferation, invasion, and colony formation, while inhibiting apoptosis in HCC cells (Figure 5A–G and S2A-D). Conversely, knockdown of WTAP in CD8^+^ T cells hindered cell proliferation, invasion, and clone formation, while promoting apoptosis (Figure 5A–G).

### 3.6. WTAP Regulated PD1 Expression in an m6A-Dependent Manner

**Figure 6:**
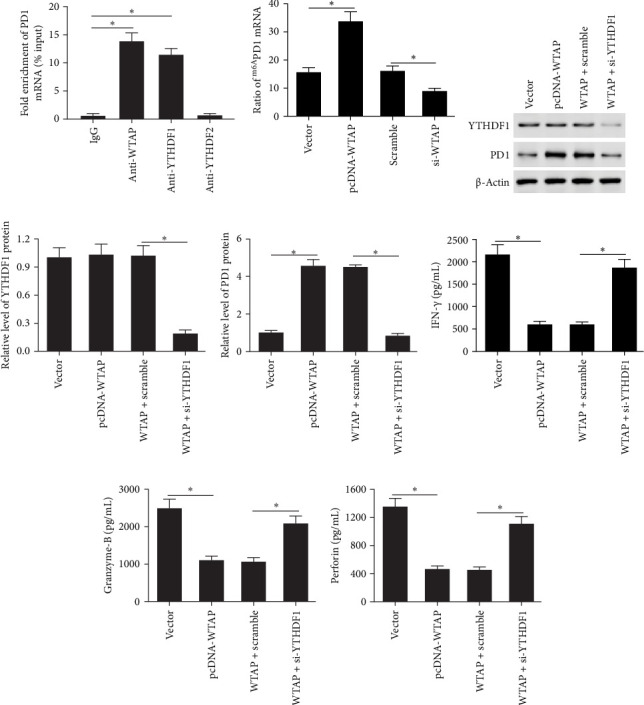
WTAP regulated PD1 expression in an m6A-dependent manner. (A) RIP assay was performed to detect the binding of WTAP, YTHDF1, and YTHDF2 with PD1 mRNA. (B) MeRIP-qPCR assay was conducted to measure the expression of m6A-modified PD1 in CD8^+^ T cells transfected with pcDNA-WTAP, WTAP siRNA, or respective controls. pcDNA-WTAP or pcDNA-WTAP + YTHDF1 siRNA were transfected into CD8^+^ T cells for 48 h. (C–E) The protein levels of YTHDF1 and PD1 were detected using western blotting. (F–H) ELISA assay was used to assess the secretion levels of IFN-γ, perforin and granzyme-B. *⁣*^*∗*^*p* < 0.05.

We first examined whether the key m6A-modifying enzymes bind to PD1 mRNA. The data showed that the m6A methyltransferases WTAP and the m6A “reader” YTHDF1, but not YTHDF2, bound to PD1 mRNA (Figure 6A). Next, we investigated whether WTAP regulates PD1 expression by modulating its m6A level. The findings indicated that overexpression of WTAP in CD8^+^ T cells increased the m6A level of PD1, whereas knockdown of WTAP had the opposite effect (Figure 6B). To determine whether YTHDF1 is involved in regulating the m6A level of PD1, we transfected pcDNA-WTAP into CD8^+^ T cells either alone or in combination with YTHDF1 siRNA. Overexpression of WTAP did not affect the protein level of YTHDF1, while transfection with YTHDF1 siRNA markedly reduced YTHDF1 expression (Figure 6C,D). Knockdown of YTHDF1 reversed the increase in the protein levels of PD1 (Figure 6C,E). Analysis of the GEPIA database and validation in HCC samples revealed that WTAP expression was significantly positively correlated with TIM3 and CTLA4, but not with LAG3 (Figure S3A-F). Overexpression of WTAP markedly increased TIM3 and CTLA4 protein levels, while leaving LAG3 unaffected (Figure S3G, H). Notably, WTAP exhibited the most pronounced upregulatory effect on PD1. Additionally, silencing YTHDF1 effectively abolished the inhibitory effect of WTAP overexpression on IFN-*γ*, granzyme-B, and perforin secretion levels (Figure 6G–I).

### 3.7. WTAP and YTHDF1 in CD8^+^ T Cells Synergistically Regulate the Malignant Behavior of HCC Cells

**Figure 7:**
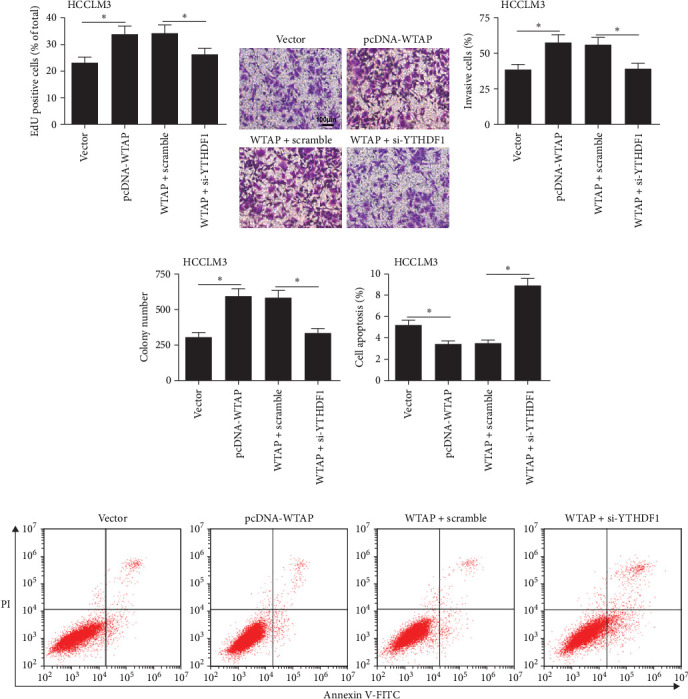
WTAP and YTHDF1 in CD8^+^ T cells synergistically regulate malignant behaviors of HCC cells. pcDNA-WTAP or pcDNA-WTAP + YTHDF1 siRNA were transfected into CD8^+^ T cells for 24 h, which were then co-cultured with HCCLM3 cells for 24 h. (A) EdU assay was performed to evaluate cell proliferation of HCCLM3 cells. (B, C) Cell invasion of HCCLM3 cells was measured using the Transwell assay. (D) Colony formation assay was conducted to assess colony formation in HCCLM3 cells. (E, F) Flow cytometry was used to analyze HCCLM3 cell apoptosis. *⁣*^*∗*^*p* < 0.05.

To further explore the effects of WTAP and YTHDF1 in CD8^+^ T cells on the behavior of HCC cells, we transfected pcDNA-WTAP either alone or together with YTHDF1 siRNA into CD8^+^ T cells, which were then co-cultured with HCCLM3 or MHCC97H cells. The data showed that silencing YTHDF1 reversed the increased cell proliferation, invasion, and clone formation, as well as the decreased apoptosis caused by WTAP overexpression in HCC cells (Figure 7A–F and Figure S4A-F).

### 3.8. WTAP Silencing Enhanced the Promotion of Anti-PD1 on the Activity and Anti-Tumor Capacity of CD8^+^ T Cells

We explored whether silencing WTAP affects the ability of anti-PD1 to enhance the activity of CD8^+^ T cells. Compared to the control group, anti-PD1 treatment significantly reduced the PD1 protein level, without altering the expression of WTAP (Figure S5A–C). In contrast, WTAP knockdown decreased the expression of both PD1 and WTAP (Figure S5A–C). Anti-PD1 treatment notably promoted CD8^+^ T cell proliferation (Figure S5D,E) and the secretion of IFN-γ, perforin, granzyme-B, and TNF-α, compared to the control group (Figure S5F–I). Interestingly, WTAP knockdown further enhanced CD8^+^ T cell proliferation and the secretion of IFN-γ and perforin, compared to the anti-PD1 group (Figure S5D–G). Next, CD8^+^ T cells treated with anti-PD1 alone or in combination with WTAP siRNA were co-cultured with HCC cells. The results showed that silencing PD1 in CD8^+^ T cells significantly inhibited cell invasion (Figure S6A–D) of HCCLM3 and MHCC97H cells, while promoting apoptosis (Figure S6E–H). Co-silencing of WTAP and PD1 in CD8^+^ T cells led to a more pronounced inhibitory effect on the malignant progression of HCC cells compared to the anti-PD1 treatment alone (Figure S6A–H).

### 3.9. Downregulation of WTAP Enhanced Anti-PD1 Mediated Tumor Inhibition in a HuNSG Mouse Model

**Figure 8:**
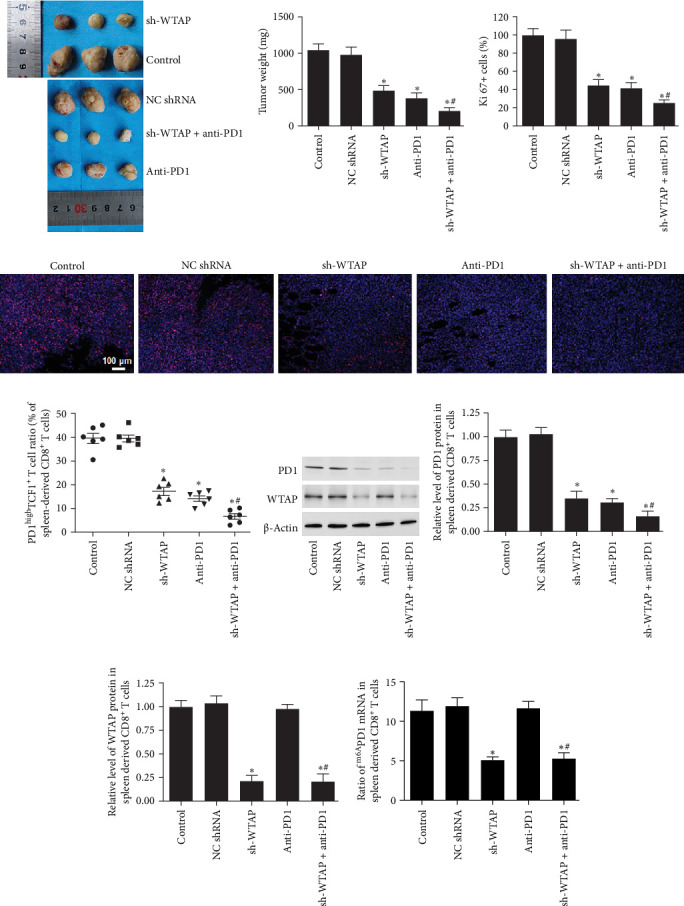
Inhibition of WTAP or PD1 impeded tumor growth in mouse xenografts. A HuNSG mouse model with a humanized immune system was constructed. Twenty-eight days after HCCLM3 cell injection, mice were sacrificed and tumor tissues and spleen tissues were isolated. (A) Representative images of tumors. (B) The weight of tumor tissues in each group was assessed. (C, D) Ki67 staining assay was used to assess tumor cell proliferation. (E) Flow cytometry was performed to evaluate the radio of PD1^high^TCF1^+^ T cells among mouse spleen CD8^+^ T cells. (F–H) The protein expression of WTAP and PD1 in mouse spleen CD8^+^ T cells was detected. (I) MeRIP-qPCR was performed to measure the level of m6A-modified PD1 in mouse spleen CD8^+^ T cells. *N* = 8. *⁣*^*∗*^*p* < 0.05 compared with the NC shRNA group. ^#^ *p* < 0.05 compared with the anti-PD1 group.

To further investigate the effect of WTAP on liver cancer progression, a humanized immune system was used to establish a HuNSG mouse model. Our data showed that silencing WTAP or treatment with anti-PD1 significantly inhibited the tumor cell proliferation and tumor growth (Figure 8A–D). The combination of WTAP silencing and anti-PD1 treatment resulted in more effective tumor growth suppression than anti-PD1 treatment alone (Figure 8A–D). WTAP knockdown or anti-PD1 treatment also reduced the proportion of PD1^high^TCF1^+^ CD8^+^ T cells in the spleen of the mice (Figure 8E). Notably, the proportion of PD1^high^TCF1^+^ CD8^+^ T cells in the WTAP silencing combined with anti-PD1 treatment group was lower than in the anti-PD1 treatment group (Figure 8E). Additionally, WTAP knockdown downregulated both WTAP and PD1 expression, as well as the PD1 m6A level in CD8^+^ T cells from the mouse spleen (Figure 8F–I). Anti-PD1 treatment significantly reduced PD1 protein levels without altering WTAP protein levels or PD1 m6A levels (Figure 8F–I). When combined, WTAP silencing and anti-PD1 treatment further reduced WTAP and PD1 expressions and PD1 m6A levels in CD8^+^ T cells from the spleen (Figure 8F–I).

## 4. Discussion

Tumor-infiltrating cytotoxic CD8^+^ T cells play a key role in suppressing tumor growth. However, persistent stimulation by cancer cells can induce a state of exhaustion or dysfunction in these cells. This is characterized by impaired proliferation, cytokine production, and cytotoxic activity, accompanied by both transcriptional and epigenetic changes [26, 27]. Two subsets of exhausted CD8^+^ T cells have been identified in the tumor immune microenvironment, distinguished by the expression of TCF1. PD1^+^ TCF1^+^ CD8^+^ T cells exhibit reduced cytotoxicity, while PD1^+^ TCF1-CD8^+^ T cells produce more cytokines and demonstrate stronger antitumor responses [28]. In recent years, immunotherapy has advanced rapidly, particularly treatments targeting the PD-1/PD-L1 pathway, which have shown significant therapeutic effects [29]. PD1, an immunosuppressive molecule expressed by activated lymphocytes, binds to PD-L1 on tumor cells to initiate programed T-cell death and trigger immune checkpoint responses [30]. Numerous studies have shown that anti-PD1 therapy can activate CD8^+^ T cells, enhancing immune responses [31, 32]. Moreover, an increasing body of evidence links the presence of circulating and tumor-infiltrating PD-1^+^ CD8^+^ T cells to poor cancer prognosis [33, 34]. A study by Ma et al. [14] highlighted that CD8^+^ PD1^high^T cells were associated with a worse prognosis in HCC patients.

Analysis using the GEPIA online database revealed that WTAP expressions did not differ significantly between normal liver and HCC tissues, nor across different stages of HCC. However, patients with high WTAP expression had a shorter MST. We isolated tumor-infiltrating CD8^+^ T cells from tumor and adjacent normal tissues and found that the proportion of PD1^high^TCF1^+^ CD8^+^ T cells was markedly higher in the tumor tissue, indicating a higher presence of functionally exhausted CD8^+^ T cells in the tumor microenvironment. Kaplan–Meier survival analysis showed that patients with a higher proportion of exhausted CD8^+^ T cells had a lower 3-year survival rate, suggesting that CD8^+^ T cell exhaustion correlates with poor prognosis in HCC patients. Furthermore, both WTAP and PD1 expression levels were elevated in tumor tissues compared to adjacent normal tissues, and these two expressions were significantly positively correlated. This suggests that high WTAP expression may be accompanied by increased PD1 expression. High expression of WTAP and PD1 was associated with advanced pathological stage in HCC, while elevated PD1 expression additionally correlated with higher histological grade. The AUC for WTAP was 0.98, demonstrating its high diagnostic accuracy in distinguishing tumor from adjacent normal tissue. In summary, WTAP may serve as a biomarker for predicting responses to PD1 therapy. This finding provides theoretical support for selecting patients who are more likely to benefit from PD1 treatment and for developing combination therapies involving WTAP and PD1 inhibitors.

The m6A modification is regulated by a network of methyltransferases, demethylases, and effector proteins. WTAP has been shown to play a significant role in various physiological and pathological processes via an m6A-dependent pathway [35]. Increasing evidence suggests that WTAP participates in tumor progression. Elevated WTAP expression in HCC is associated with poor prognosis. WTAP accelerates tumor growth in HCC xenograft models by regulating the m6A modification of ETS1 [24]. It also protects hepatoma cells from autophagic cell death by mediating the m6A modification of LKB1 [36]. Additionally, m6A regulatory factors are thought to function as key regulators in the expression of PD-L1 and immune cell infiltration, influencing the immune microenvironment in esophageal squamous cell carcinoma [37]. In pancreatic ductal adenocarcinoma, WTAP is linked to CD8^+^ T cell infiltration and positively correlates with inflammatory gene expression profiles and various immune checkpoints [38]. Our study demonstrated that WTAP inhibited CD8^+^ T cell proliferation and immune activity by promoting PD1 expression. In the CD8^+^ T-HCC cell co-culture system, silencing WTAP in CD8^+^ T cells effectively suppressed HCC cell growth and invasion. Moreover, combined silencing of WTAP and anti-PD1 treatment further enhanced CD8^+^ T cell immune activity, strengthening the suppression of HCC cell progression. In the HuNSG xenograft model, knockdown of WTAP or PD1 significantly reduced CD8^+^ T cell exhaustion and inhibited tumor progression. Notably, simultaneous silencing of both WTAP and PD1 resulted in a more pronounced antitumor effect. These findings indicate that WTAP may serve as a novel therapeutic target, enhancing the efficacy of anti-PD1 therapy by modulating immune cell function and providing new strategies for more precise immunotherapy.

The m6A “readers”, primarily composed of the YTH family, regulate the stability and translation efficiency of m6A-modified mRNAs [24, 39]. A previous study has shown that YTHDF1 facilitates the translation of m6A-modified FOXM1 to enhance breast cancer metastasis [40]. It also enhances the stem cell-like properties of cisplatin-resistant ovarian cancer cells by recognizing m6A sites on TRIM29 mRNA [41]. Furthermore, low expression of YTHDF1 correlates with improved survival in HCC patients [42]. The results in this study indicated that WTAP upregulates the m6A level of PD1 mRNA and enhances its translation via YTHDF1. The analysis of the GEPIA database and validation in HCC samples revealed that WTAP expression showed significant positive correlations with other immune checkpoint molecules (TIM-3 and CTLA-4), and WTAP overexpression upregulated TIM-3 and CTLA-4 protein levels. However, whether WTAP promotes CD8^+^ T cell exhaustion through immune checkpoint molecules other than PD-1 remains to be further investigated. Moreover, we found that silencing YTHDF1 reversed the inhibitory effect of WTAP overexpression on CD8^+^ T cell proliferation and immune function, as well as the promotion of PD1 expression by WTAP. Additionally, in the CD8^+^ T-HCC cell co-culture system, silencing YTHDF1 antagonized the role of WTAP in promoting the malignant progression of HCCLM3 and MHCC97H cells.

## 5. Conclusions

This study demonstrated that WTAP, as an m6A methyltransferase, promoted the m6A modification of PD1 and enhanced its translation through YTHDF1. By regulating PD1 expression, WTAP suppressed the immune activity of CD8^+^ T cells, thereby facilitating the progression of HCC cells in the CD8^+^ T-HCC cell co-culture system. Additionally, WTAP knockdown not only alleviated CD8^+^ T cell exhaustion and inhibited tumor progression in HuNSG xenograft mice but also synergistically enhanced the antitumor efficacy of anti-PD1 therapy. These findings suggest that targeting WTAP could serve as a promising immunotherapeutic strategy for HCC patients.

## Figures and Tables

**Table 1:** Correlation between WTAP expression and different clinicopathological features.

Characteristic	Low expression of WTAP	High expression of WTAP	*p*-Value
*N*	62	62	—
Age, *n* (%)	—	—	0.590
≤60	32 (25.8%)	28 (22.6%)	—
>60	30 (24.2%)	34 (27.4%)	—
Gender, *n* (%)	—	—	0.719
Female	33 (26.6%)	30 (24.2%)	—
Male	29 (23.4%)	32 (25.8%)	—
T stage, *n* (%)	—	—	0.360
T1	39 (31.4%)	30 (24.2%)	—
T2	13 (10.5%)	16 (12.9%)	—
T3	9 (7.3%)	13 (10.5%)	—
T4	1 (0.8%)	3 (2.4%)	—
N stage, *n* (%)	—	—	0.315
N0	62 (50.0%)	61 (49.2%)	—
N1	0 (0.0%)	1 (0.8%)	—
M stage, *n* (%)	—	—	1.000
M0	61 (49.2%)	61 (49.2%)	—
M1	1 (0.8%)	1 (0.8%)	—
Pathologic stage, *n* (%)	—	—	0.023
Stage I	21 (16.9%)	24 (19.3%)	—
Stage II	25 (20.2%)	12 (9.7%)	—
Stage III	13 (10.5%)	25 (20.2%)	—
Stage IV	3 (2.4%)	1 (0.8%)	—
Histologic grade, *n* (%)	—	—	0.567
G1	14 (11.3%)	8 (6.5%)	—
G2	29 (23.4%)	32 (25.8%)	—
G3	17 (13.7%)	20 (16.1%)	—
G4	2 (1.6%)	2 (1.6%)	—
Ablation embolization, *n* (%)	—	—	0.559
No	61 (49.2%)	60 (48.4%)	—
Yes	1 (0.8%)	2 (1.6%)	—
Postoperative radiotherapy, *n* (%)	—	—	0.697
No	58 (46.8%)	59 (47.6%)	—
Yes	4 (3.2%)	3 (2.4%)	—
Cancer status, *n* (%)	—	—	0.369
Tumor free	35 (28.2%)	29 (23.4%)	—
With tumor	27 (21.8%)	33 (26.6%)	—

## Data Availability

The data supporting the findings of this study are available from the corresponding author upon reasonable request.

## Supporting Information

Supporting Information The following supporting figures are available with this article: Figure S1: Demographic associations and CD8^+^ T cell subset distribution of WTAP and PD-1 in HCC. Figure S2: Overexpression of WTAP in CD8^+^ T cells promoted cell invasion and reduced apoptosis in MHCC97 cells. Figure S3: Correlation analysis between WTAP and immune checkpoint molecule expression levels. Figure S4: WTAP and YTHDF1 in CD8^+^ T cells synergistically regulate malignant behaviors of MHCC97H cells. Figure S5: WTAP silencing enhanced the promotion of anti-PD1 on the activity of CD8^+^ T cells. Figure S6: WTAP silencing enhanced the promotion of anti-PD1 on the antitumor capacity of CD8^+^ T cells.
