# Supplementary material for: WTAP Accelerates Exhaustion of CD8+ T Cells and Progression of Hepatocellular Carcinoma by Promoting m6A Modification and Translation of PD1 mRNA
Source: Mediators Inflamm. 2025 Jun 18;2025:6217272. doi: 10.1155/mi/6217272 (PMC12197553; doi:10.1155/mi/6217272)
Supplement: Supporting Information — The following supporting figures are available with this article: Figure S1: Demographic associations and CD8+ T cell subset distribution of WTAP and PD-1 in HCC. Figure S2: Overexpression of WTAP in CD8+ T cells promoted cell invasion and reduced apoptosis in MHCC97 cells. Figure S3: Correlation analysis between WTAP and immune checkpoint molecule expression levels. Figure S4: WTAP and YTHDF1 in CD8+ T cells synergistically regulate malignant behaviors of MHCC97H cells. Figure S5: WTAP silencing enhanced the promotion of anti-PD1 on the activity of CD8+ T cells. Figure S6: WTAP silencing enhanced the promotion of anti-PD1 on the antitumor capacity of CD8+ T cells. [file 6217272.f1.docx]

**
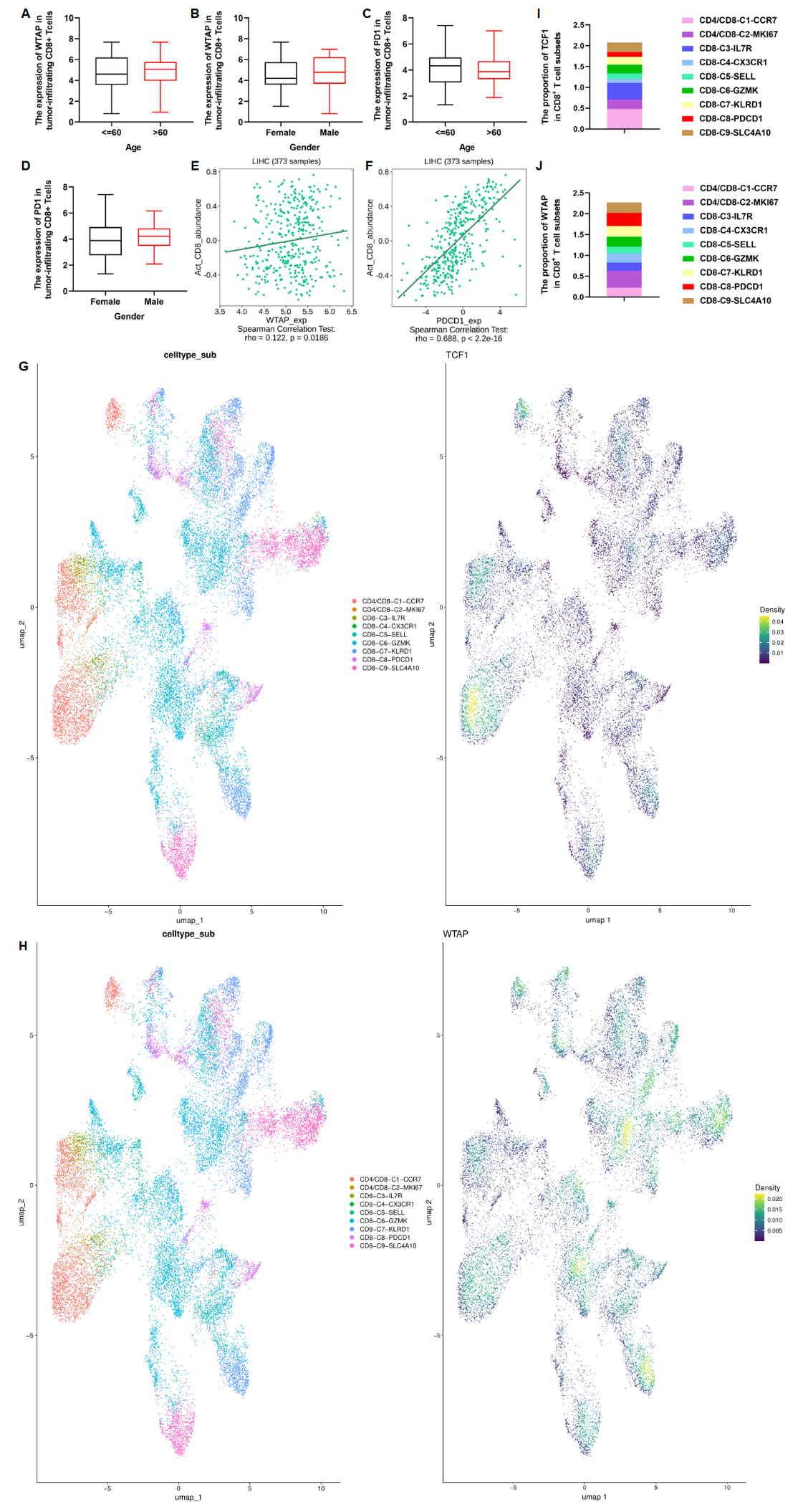
**

**Supplementary figure 1.** (A-D) Expression of WTAP and PD-1 in different clinical subtypes of HCC samples (N=124). (E, F) The TISIDB database was utilized to assess the correlation between WTAP/PD-1 expression levels and the abundance of activated CD8^+^ T cells in liver cancer (N=373). (G, H) The left panels of Figures G and H demonstrate the proportional distribution of distinct CD8^+^ T cell subsets within the HCC tumor microenvironment. The right panel of Figure G specifically illustrates the prevalence of TCF1 expression across these CD8^+^ T cell subpopulations, while the right panel of Figure H correspondingly displays the distribution pattern of WTAP expression among the subsets. (I-J) Figures I and J display the quantitative analysis of TCF1 and WTAP expression frequencies among various CD8^+^ T cell subpopulations.


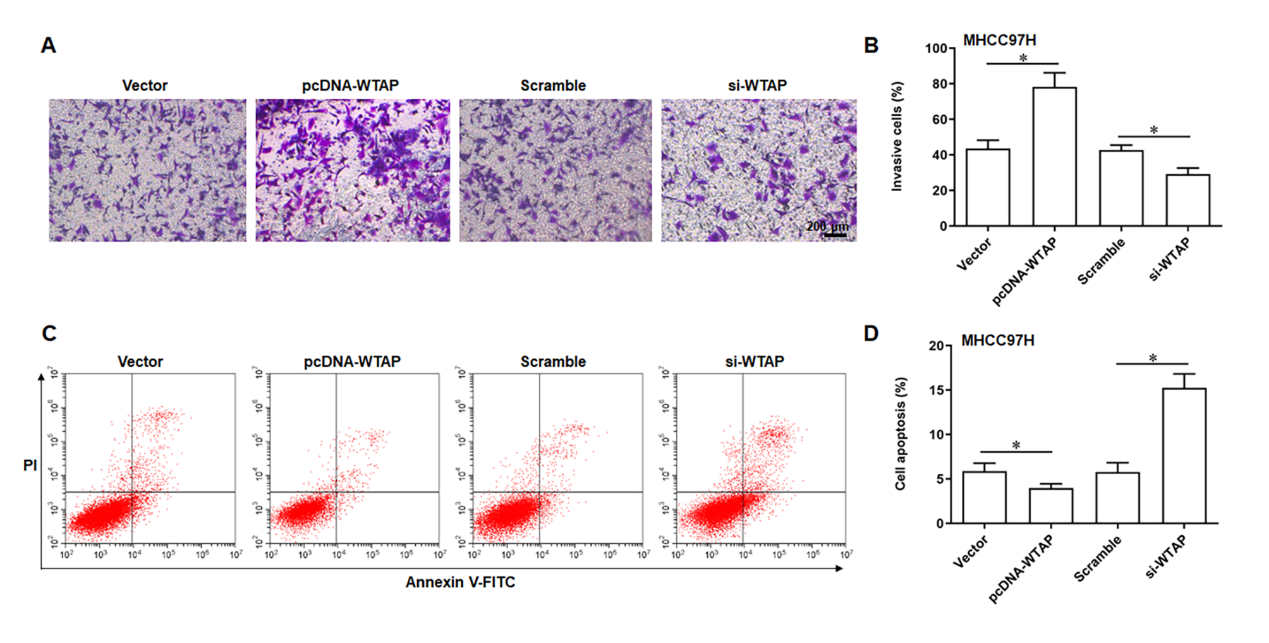


**Supplementary figure 2.** Overexpression of WTAP in CD8^+^ T cells promoted cell invasion and reduced apoptosis in MHCC97 cells. pcDNA-WTAP or WTAP siRNA were transfected into CD8^+^ T cells for 24 h, which were then co-cultured with MHCC97H cells for 24 h. (A, B) Cell invasion of MHCC97H cells was analyzed using Transwell assay. (C, D) Flow cytometry was performed to detect MHCC97H cell apoptosis. **P*<0.05.


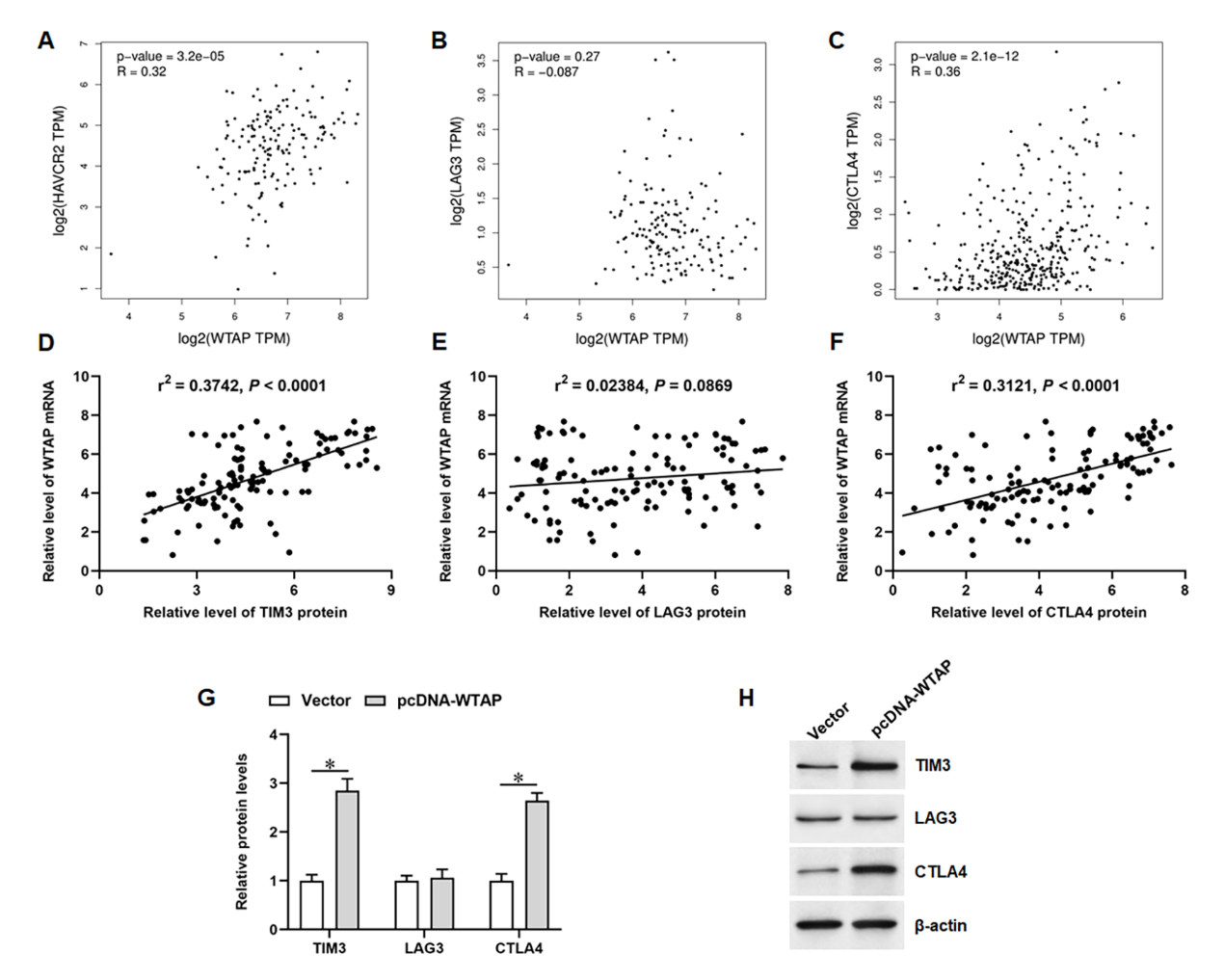


**Supplementary figure 3.** Correlation analysis between WTAP and immune checkpoint molecule expression levels. (A-C) GEPIA database analysis was performed to assess associations of WTAP with TIM-3, LAG-3, and CTLA-4 expression in liver cancer. (D-F) The association between WTAP expression and TIM-3, LAG-3, or CTLA-4 levels was assessed using Pearson correlation analysis. (G, H) pcDNA-WTAP was transfected into CD8^+^ T cells for 48 h, and the protein levels of TIM-3, LAG-3, or CTLA-4 were detected using Western blotting. **P*<0.05.


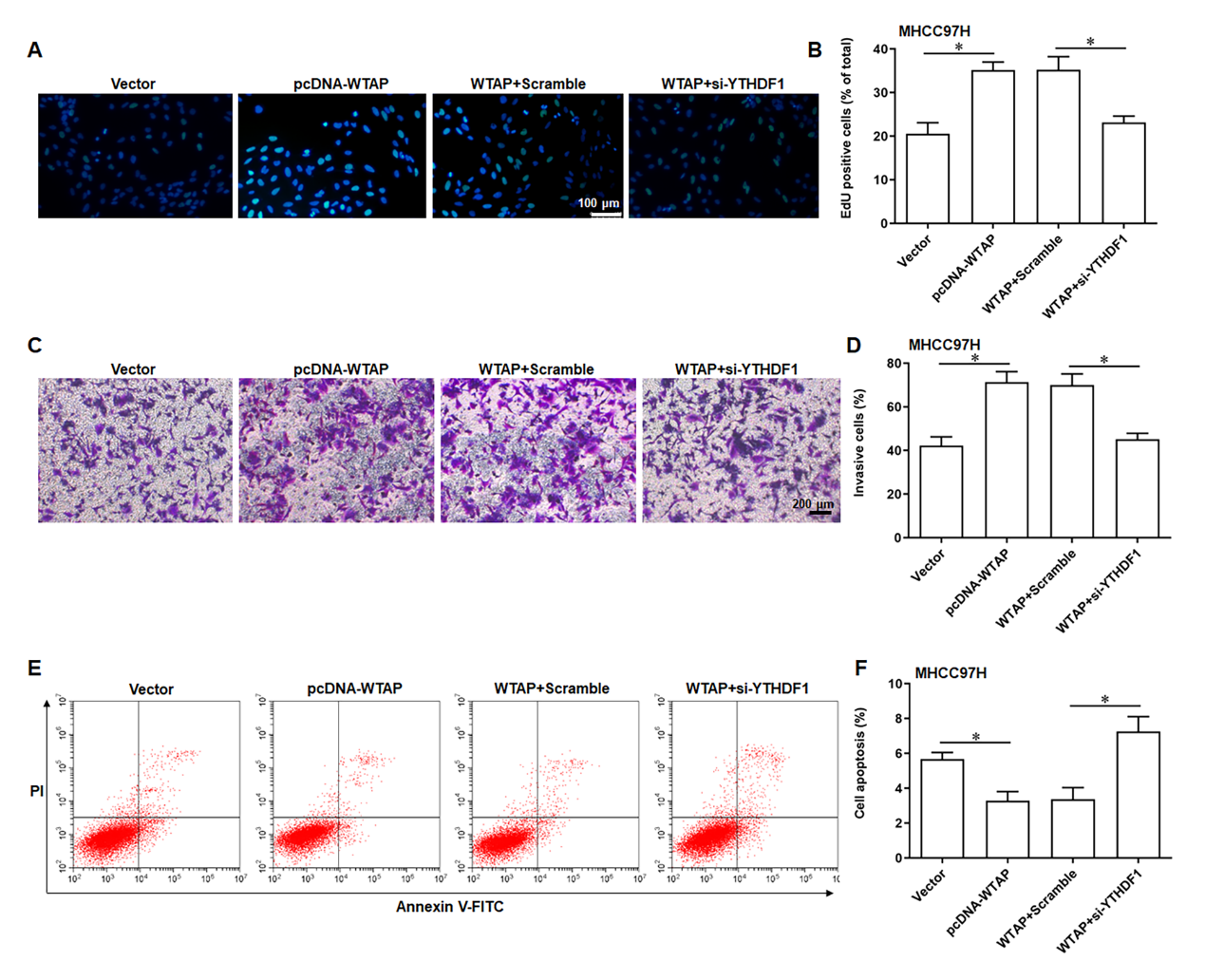


**Supplementary figure 4.** WTAP and YTHDF1 in CD8^+^ T cells synergistically regulate malignant behaviors of MHCC97H cells. pcDNA-WTAP or pcDNA-WTAP + YTHDF1 siRNA were transfected into CD8^+^ T cells for 24 h, which were then co-cultured with MHCC97H cells for 24 h. (A, B) EdU assay was performed to evaluate cell proliferation of MHCC97H cells. (C, D) Cell invasion of MHCC97H cells was measured using Transwell assay. (E, F) Flow cytometry was used to analyze MHCC97H cell apoptosis. **P*<0.05.


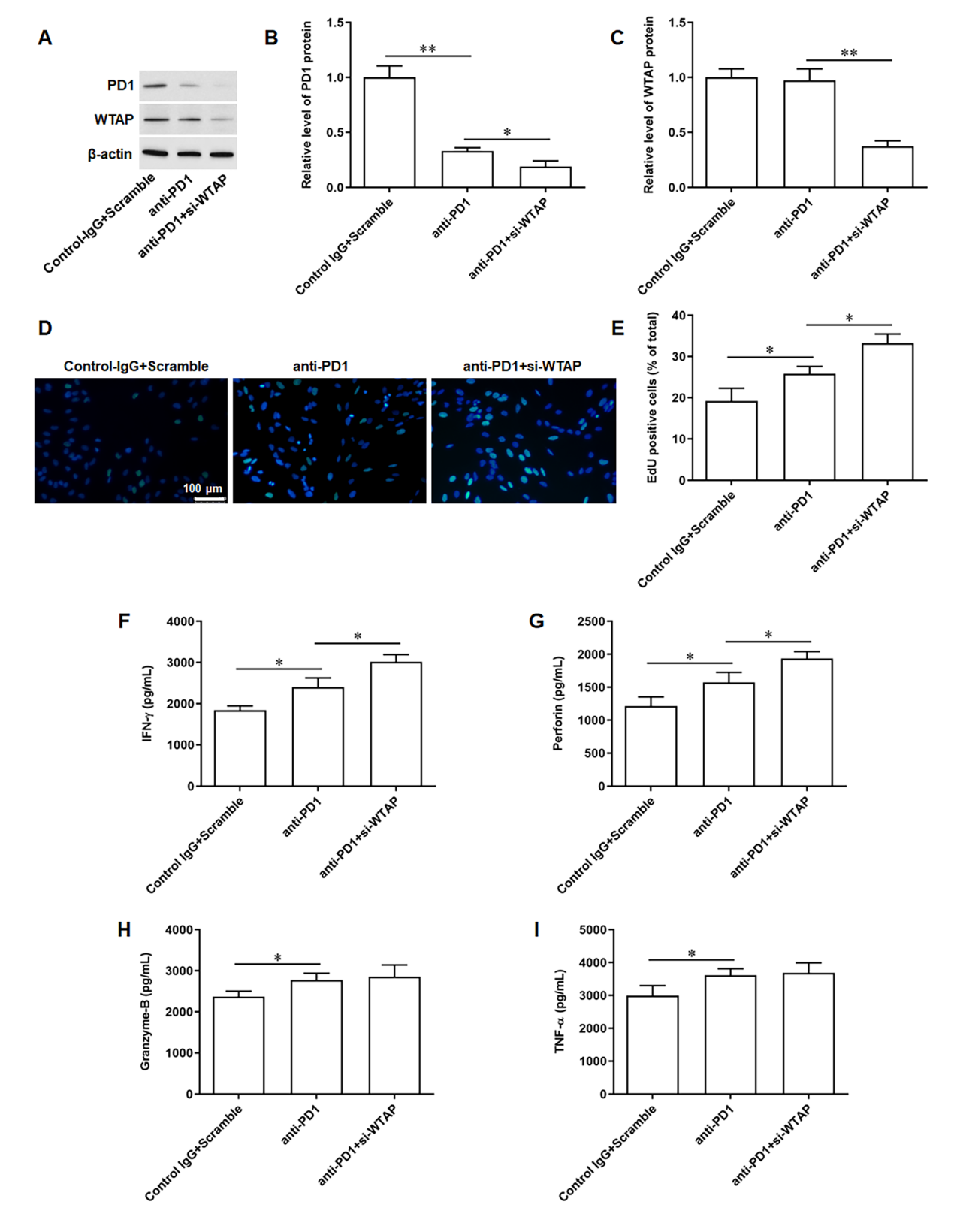


**Supplementary figure 5.** WTAP silencing enhanced the promotion of anti-PD1 on the activity of CD8^+^ T cells. CD8^+^ T cells were treated with anti-PD1 alone or co-treated with anti-PD1 and WTAP siRNA. (A-C) The protein levels of PD1 and WTAP in CD8^+^ T cells were detected using Western blotting. (D, E) EdU staining was used to assess cell proliferation of CD8^+^ T cells. (F-I) ELISA was performed to analyze the levels of TNF-α, IFN-γ, Perforin and Granzyme-B. **P*<0.05, ***P*<0.01.


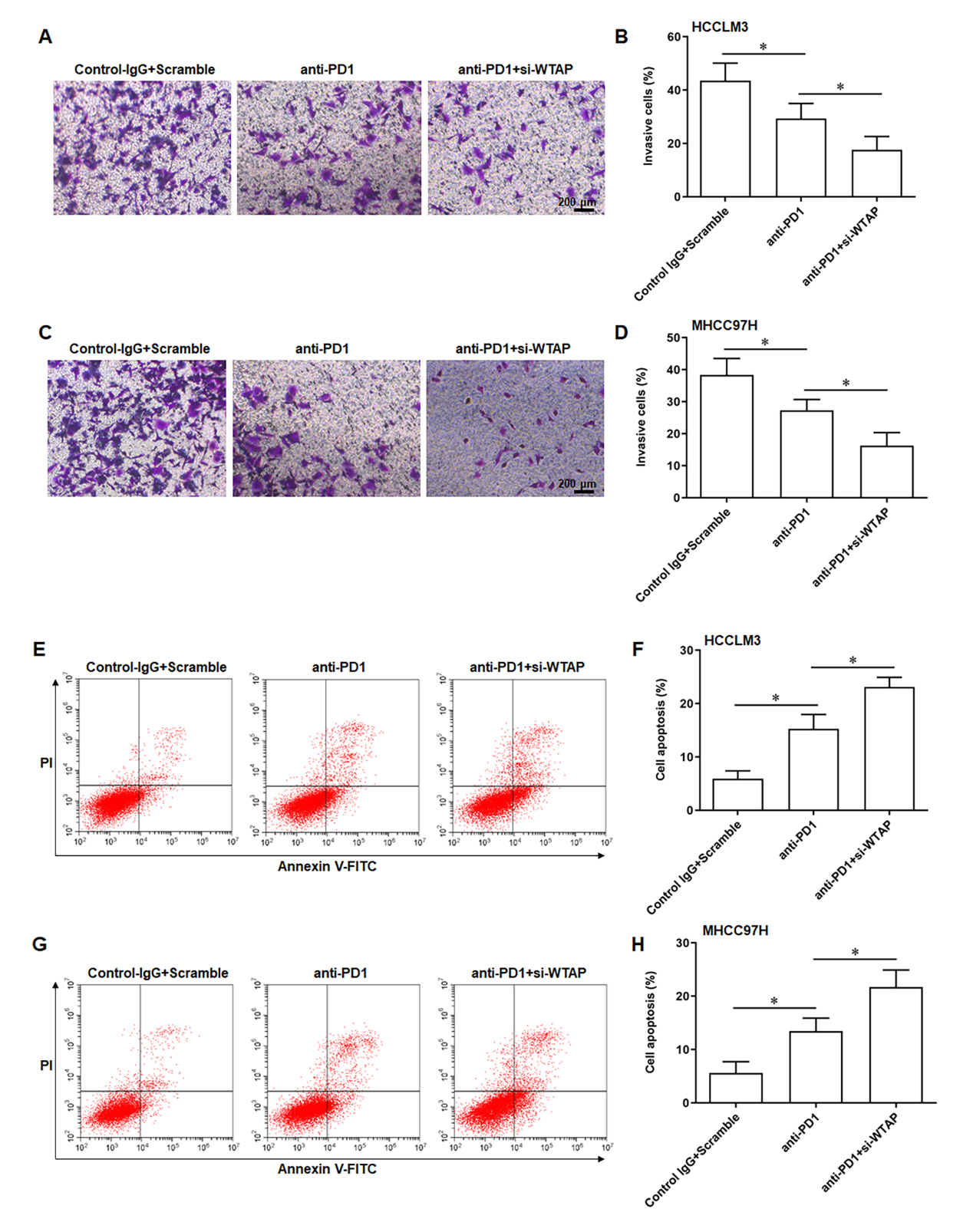


**Supplementary figure 6.** WTAP silencing enhanced the promotion of anti-PD1 on the anti-tumor capacity of CD8^+^ T cells. CD8^+^ T cells treated with anti-PD1 alone or together with WTAP siRNA were co-cultured with HCCLM3 or MHCC97H cells for 24 h. (A-D) Cell invasion of HCCLM3 and MHCC97H cells was measured using Transwell assay. (E-H) Cell apoptosis of HCCLM3 and MHCC97H cells were assessed by Flow cytometry. **P*<0.05.
